# Creation of an ultra scale‐down bioreactor mimic for rapid development of lignocellulosic enzymatic hydrolysis processes

**DOI:** 10.1002/jctb.4801

**Published:** 2015-09-15

**Authors:** Neil Conroy, Ian Tebble, Gary J Lye

**Affiliations:** ^1^ ReBio Technologies LtdUnit 59 Dunsfold ParkCranleighSurreyGU6 8TBUK; ^2^ The Advanced Centre for Biochemical Engineering, Department of Biochemical EngineeringUniversity College LondonGordon StreetLondonWC1H 0AHUK

**Keywords:** distillers' dried grains with solubles, bioethanol, enzyme hydrolysis, scale down

## Abstract

**BACKGROUND:**

Cellulosic bioethanol processes involve several steps, all of which require experimental optimisation. A significant aid to this research would be a validated ultra scale‐down (USD) model that could be used to perform rapid, wide ranging screening and optimisation experiments using limited materials under process relevant conditions.

**RESULTS:**

In this work, the use of 30 mL shaken conical tubes as a USD model for an enzymatic hydrolysis process is established. The approach is demonstrated for the hydrolysis of distillers' dried grains with solubles (DDGS). Results from the USD tubes closely mimic those obtained from 4 L stirred tanks, in terms of the rate, composition and concentrations of sugars released, representing an 80‐fold scale reduction. The utility of the USD approach is illustrated by investigating factors that may be limiting hydrolysis yields at high solids loadings. Washing the residual solids periodically during hydrolysis allowed 100% of the available sugar to be hydrolysed using commercially available enzymes.

**CONCLUSION:**

The results demonstrate that the USD system reported successfully mimics the performance of conventional stirred tanks under industrially relevant conditions. The utility of the system was confirmed through its use to investigate performance limitation using a commercially relevant feedstock. © 2015 The Authors. *Journal of Chemical Technology & Biotechnology* published by John Wiley & Sons Ltd on behalf of Society of Chemical Industry.

## INTRODUCTION

Large parts of the world's population are currently dependent on fossil fuels for personal transportation.^1^ Concern about the impact greenhouse gas emissions from these fuels have on global climate change and predictions that world oil production may reach a peak in the near future have prompted governments and researchers to seek alternatives.^2^ Currently the main alternative cited is first generation bioethanol which is produced from starch or sugar crops, with 100 billion litres being produced annually.^3^ It is however recognised that producing bioethanol in this way is limited in the long‐term due to land use requirements and competition with food production.

Second generation bioethanol processes utilise the sugars contained within the lignocellulosic structure of plants. This creates the opportunity to use waste sources such as agricultural residues, forestry off cuts and municipal solid waste to produce large quantities of bioethanol.^4^ Lignocellulose is a complex structural macromolecule that can contain up to 80% (w/w) sugars along with lignin.^5^ The sugars in lignocellulose are primarily present as the polymers cellulose and hemicellulose as opposed to the simple sugars or starch used in current bioethanol production processes.^6^ Lignocellulose is naturally resistant to breakdown and so proposed lignocellulosic ethanol processes tend to be more complicated than starch or sugar based processes.

Most currently envisioned second generation processes involve three main process steps: pretreatment, enzymatic hydrolysis and fermentation. The pretreatment step is used primarily to open up the structure of the lignocellulose fibres and increase their accessibility to enzymes. Depending on the method used some sugars may also be solubilised at this stage. A number of different pretreatment methods and technologies, most of them physio‐chemical, have been proposed and have been extensively reviewed elsewhere.^7^, ^8^ In the subsequent enzyme hydrolysis step a complex mixture of cellulolytic enzymes is added to the pretreated biomass in order to convert the insoluble cellulose and hemicellulose to soluble short chain or monomeric sugars. In the fermentation stage these sugars are converted into ethanol by a fermentative microorganism. Traditional *Saccharomyces cerevisiae* strains are not well suited to this process due to their limited substrate range leading to a variety of possible alternatives being used. These include both *S. cerevisiae* strains engineered to have a wider substrate range and novel alternative organisms.^9^, ^10^

The enzymatic hydrolysis is a key step in the overall process yet it is comparatively poorly understood due to the complex nature of the substrates and enzymes. Both the absolute concentration of sugar in a feedstock and the relative proportions of the different sugars vary considerably according to its type and source. In addition the different pretreatment options available have substantially different effects on the biomass with some solubilising very few sugars while others solubilise a substantial portion of the hemicellulose.^11^ Likewise, commercially available cellulolytic enzymes are complex mixtures with multiple complimentary activities.^12^, ^13^ These factors severely limit any attempts to develop predictive mathematical models of the process, meaning researchers have to rely on a large number of experiments to inform process development.^14^

This need for these scouting and optimisation experiments means that there is significant interest in the use of ultra scale‐down (USD) models to speed up process development.^15^ Such techniques are well established in the pharmaceutical sector with reports of successful transfers from scale‐down devices to bioreactors orders of magnitude larger.^16^, ^17^ Early reports of the utilisation of microtiter plates with lignocellulosic slurries for the enzymatic hydrolysis or fermentation process stages required modification of the biomass to render it more amenable for use at very small scales.^13^, ^18^ Methods have also been developed to carry out the pretreatment stage at microtiter scale, allowing integrated pretreatment and enzyme hydrolysis trials.^19^ Extensive milling of the biomass is currently required for all of these methods. More recently methods have been developed that reduce or remove the need for particle size reduction.^20^ These methods are well suited to initial screening experiments however to date no kinetic data has been published showing comparability with conventional STRs. Such information is particularly important for process development in industry and thus there remains a requirement for a small scale, low cost system that can accurately replicate the kinetics of processes at STR scales.

In this work the use of shaken, small scale tube‐based devices as USD models suitable for the direct investigation of lignocellulosic slurry hydrolysis at high solids loading is reported. Distillers' dried grains with solubles (DDGS) was chosen as a realistic feedstock as it has been characterised extensively by other groups and presents an opportunity to increase the efficiency of existing corn ethanol by utilising a by‐product of limited value to produce additional ethanol.^21^, ^22^ The results demonstrate that in terms of both the final yield and the process kinetics the USD tubes accurately reflect the performance seen in conventional stirred tank reactors (STRs). The USD tubes were additionally used to investigate the factors limiting process performance, confirming that soluble inhibitors have a significant negative effect on process performance. Thus a scale down system capable of accurately mimicking STR performance using unmodified slurries at a much reduced scale is described.

## MATERIALS AND METHODS

### Materials

Distillers' dried grains with solubles (DDGS) was purchased from Trident Feeds (Cambridgeshire, UK). The moisture content was analysed using standard National Renewable Energy Laboratory (NREL) techniques and was determined to be 10.2% (w/w).^23^ All chemicals used in this work were of analytical grade unless otherwise stated and were sourced from Sigma Aldrich (Poole, Dorset, UK) or Fisher Scientific (Loughborough, UK). The supply of the enzymes used is covered by non‐disclosure agreements with commercial suppliers and so they will simply be referred to as A, B and C. Enzymes A and B are liquid mixtures containing mainly cellulases supplemented with hemicellulases. Enzyme C is a food processing aid that contains mainly hemicellulases and was supplied in the form of a dry powder and so was prepared as a 20% (w/w) stock solution prior to use. Stock solutions were stored for up to 1 week and then discarded. Enzymes A and B were used at a loading of 3.33 mL kg^−1^ dry DDGS and enzyme C was added at 5 g kg^−1^ dry DDGS. This loading was determined to be the optimal commercial loading based on confidential price information from suppliers and ReBio process–economic models and was consequently used for all experimental work.

### Pretreatment of DDGS

Slurries of DDGS were pretreated in a custom built, pilot scale steam explosion reactor with a total volume of 68 L and multiple steam injection nozzles. Appropriate amounts of DDGS were weighed out (correcting for the moisture content of the DDGS) and then diluted with hot water until the desired level of dry solids, 30% (w/w), was reached. All dry solids loadings, abbreviated to %DS, in this work are given as the percentage by mass of bone dry solids in a slurry. The slurry was then mixed thoroughly before being transferred into the pretreatment reactor. Dry saturated steam was added to the reactor to reach a pressure of 6.5 bar and maintain the contents at this pressure for 5 min. After the hold time was completed the steam valve was opened again, and the pressure was increased to 20 bar. At this point the steam valve was closed and immediately afterwards a discharge valve located at the bottom of the reactor was opened and the material was discharged into a second vessel at atmospheric pressure. This material was then well‐mixed and discharged into suitable 5 L containers to be stored at −20°C. Pretreated DDGS was stored for a maximum of 1 month, after which time fresh material was generated.

### Enzymatic hydrolysis in STRs

Tank hydrolyses were carried out in 4 L working volume (7.5 L total volume) bioreactors (Biostat CT‐DCU‐5‐2, Sartorius UK). The agitator shaft was fitted with two Rushton turbine impellors (d_i_ = 65 mm), spaced equidistantly on the shaft up to 10 mm below the fill level, stirring at 200 rpm. Sodium azide at 0.04% (w/v) was used to control microbial contamination. Slurries were prepared from DDGS that had been pretreated as described and diluted with an appropriate volume of water to reach the desired dry solids content. Slurries were then adjusted to pH 5.0 ± 0.1 with 32% v/v NaOH (Brentag, Leeds, UK) before addition of the enzymes set out above.

### Enzymatic hydrolysis in USD tubes

Slurries of pretreated DDGS were prepared by diluting pretreated feedstock with an appropriate quantity of water. The pH of the slurry was adjusted to 5.0 ± 0.1 and 0.04% (w/v) sodium azide was added. Hydrolysis was carried out in 50 mL conical bottom polypropylene tubes (Starlab, Milton Keynes, UK) filled with 30 ± 0.1 g of slurry and warmed in an incubator set to 50°C for 1 h before the addition of enzymes. Following enzyme addition, the tubes were vigorously mixed by hand and then placed into an incubator shaker (Sartorius Certomat BS‐1, Sartorius UK) with a 50 mm orbital diameter that was set at 50°C and 250 rpm shaking frequency for the desired hydrolysis time. In order to generate time course data for tube hydrolysis, multiple tubes were started at the same time, with sacrificial tubes being removed at desired intervals. For all tube experiments, each condition was run either in duplicate or triplicate. Unless otherwise noted, all hydrolyses were run with the ‘1×’ enzyme loading, as set out above, added in one dose at the beginning of the hydrolysis period.

### Imaging of mixing in USD tubes

Images were recorded using a Fastcam DVR high speed video recorder (Photron Europe Limited, West Wycombe, UK). The tubes were held on an orbital shaking platform using a combination of clamps that allowed the angle of the tube to be varied without impeding the field of vision of the camera. Images were recorded at 500 frames per second at various shaking frequencies. The tubes were filled with hydrolysed DDGS and the images produced from the recordings were stored for later use and analysis.

### Enzyme hydrolysis with washed solids

For these experiments, hydrolysis was started in USD tubes and at certain time points the tubes were removed from the shaker and centrifuged to separate the solids (3820 g, 5 min). The supernatant was poured off, weighed and then stored for further analysis. The solids were re‐suspended in 30 mL of water, mixed well and then centrifuged; again the supernatant was weighed and stored. The pellet was re‐suspended in 0.5 mol L^−1^ citrate buffer (pH 5.0) to a total of 30 g and then fresh enzyme was added and the tubes returned to the shaker. The study is summarized in Table 1.

**Table 1:** Frequency and interval of wash steps during the sequential hydrolysis of DDGS. Hydrolyses carried out in USD tubes at initial solids loading of 20% (w/w)

	Control	1 wash	2 washes	3 washes
Initial enzyme loading	2×	1×	1×	1×
Wash 1 time	‐‐‐	8 h	4 h	2 h
Restart 1 enzyme loading	‐‐‐	0.5×	0.5×	0.25×
Wash 2 time	‐‐‐	‐‐‐	8 h	4 h
Restart 2 enzyme loading	‐‐‐	‐‐‐	0.5×	0.25×
Wash 3 time	‐‐‐	‐‐‐	‐‐‐	8 h
Restart 3 enzyme loading	‐‐‐	‐‐‐	‐‐‐	0.5×
Total hydrolysis time	24 h	24 h	24 h	24 h

### Determination of sugar concentrations

All samples were centrifuged in order to remove solids, and the supernatant passed through a 0.2 µm nylon membrane filter (PEHNEX NY, Phenomenex, Macclesfield, UK) to remove any particulates. Sugars were determined by HPLC (Alliance 2695, Waters, Elstree, UK) using a Rezex RHM column (7.8 × 300 mm, 8 µm packing) (Phenomenex, UK) with a 4 mmol L^−1^ H_2_SO_4_ mobile phase at a flow rate of 0.6 mL min^−1^. Detection of compounds was by UV adsorption (Waters 2996, Waters, UK) and differential Refractive Index (RI) (Waters 2414, Waters, UK) detectors operated in series. Concentrations of glucose, xylose, galactose, mannose, fructose, cellobiose and arabinose were determined using calibration curves prepared with standards of known concentration. Using this method, xylose, mannose, galactose and fructose co‐elute and so cannot be separated. The concentrations of these four sugars are therefore reported combined as X + G + M + F. In DDGS this peak will mainly represent xylose as only small amounts of the other sugars are present.

Oligomeric sugars were monomerised by heating to 121°C for 60 min in the presence of 4% (w/w) H_2_SO_4_. Sugar concentrations were then determined as for the monomers, and the concentration of oligomers calculated by subtracting the monomer concentration of the sample prior to the acid treatment.

Hydrolysis progress was compared by calculating a hydrolysis yield, dividing the concentration of a sugar(s) in the hydrolysate by the maximum concentration of that sugar(s) that could be achieved given the amount and composition of feedstock in the hydrolysate.

## RESULTS AND DISCUSSION

### Rationale for the selection of the ultra scale down tubes

In the establishment of USD approaches to industrial lignocellulosic hydrolysis development there are several key requirements that need to be met. Briefly, any device must be of a small scale (ideally less than 100 mL in volume) and with a small footprint to minimise material use and maximise the number of experiments carried out simultaneously. In order to enhance their utility they should use pretreated lignocellulose slurries without modification. Finally, the results obtained from the USD tubes need to be reproducible across experiments and be capable of accurately replicating results from larger scales.

The selection of 50 mL conical bottom tubes for use as a USD model was a compromise between the different criteria. Microwell plates would reduce material use further and offer greater opportunities for experimental automation but generally require particle size reduction which is considered undesirable. The very small volumes used (<5 mL) could increase experimental error especially with non‐homogenous slurries. Shake flasks have often been used for lignocellulosic hydrolysis experiments and the larger quantities of material used should improve reproducibility since a major source of error when working with slurries is their non‐homogenous nature. However, shake flasks do not offer particularly large reductions in material or space requirements relative to STRs. Conical tubes offered the best compromise between these two options. A rack for 16 conical tubes occupies the same space on a shaker platform as four 500 mL shake flasks and thus a four‐fold increase in experimental throughput. The quantities of biomass, enzymes and other reagents required will be likewise reduced.

Similar tubes have previously been used for suspension culture of mammalian cells^24^ however the dry solids fraction was negligible and the cells were small, 10–20 µm, with a density close to that of water. The current application is considerably more challenging with the requirement to work at dry solids fractions up to 25% (w/w) and unmilled DDGS with particle sizes in excess of 5 mm. Similar tubes have also previously been used for the experimental validation of enzyme hydrolysis yield calculations.^25^ There is, however, a lack of published work providing a detailed characterisation of such a system when used with lignocellulosic materials and there is a need for detailed process kinetics to be determined and compared with more established systems such as stirred bioreactors.

Initial experiments were therefore performed to visually assess the efficiency of solids suspension in the USD tubes at various dry solids levels when orbitally shaken at a range of frequencies. These indicated that good solids suspension could be achieved working with a mass of 30 g of whole pretreated DDGS slurry shaken at 250 rpm. These conditions were used in the subsequent investigations

### Kinetics of sugar solubilisation in a 4 L STR

Distillers' dried grains with solubles (DDGS) is a by‐product of the production of ethanol from corn in first generation processes and is primarily utilised as an animal feed. After volatile components are removed from the fermentation broth the remaining liquid passes through a centrifuge to separate the solids. These solids can either be utilised as is, ‘wet cake’, or can be combined with the supernatant from the centrifuge after it has been significantly concentrated. The recombined process stream then passes through a drying step to produce DDGS. As much of the value of DDGS is primarily due to its protein content, the sugars present in it could be utilised to produce more ethanol and still leave a protein‐rich animal feed by‐product at the end of the process 21.

**Figure 1:**
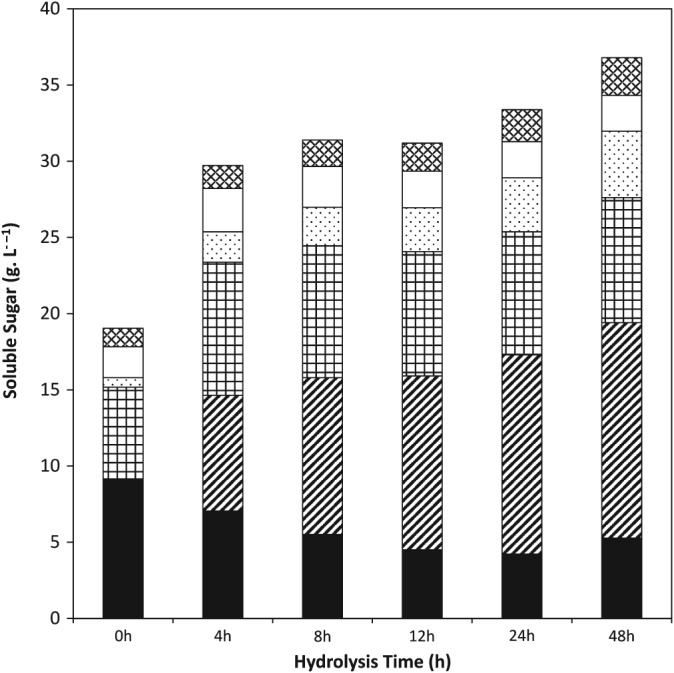
Kinetics of sugar solubilisation during hydrolysis of DDGS at 10% (w/w) total solids loading in a conventional 4 L STR: (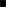 **)** glucose oligomers; (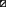) monomeric glucose; (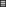) xylose oligomers; (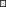) xylose monomer; (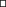) arabinose oligomers; (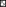) arabinose monomer.

Figure 1 shows that the enzymatic hydrolysis of the DDGS slurry in a conventional lab scale STR, following thermal pretreatment, is a rapid process. 85% (w/w) of the sugar that was solubilised by the end of the enzyme hydrolysis was released within the first 8 h, a substantial proportion of this during pretreatment. Over the first 8 h of hydrolysis sugar was solubilised at a rate in excess of 1.5 g L^−1^ h^−1^, whereas the rate over the remaining 40 h of hydrolysis was 0.13 g L^−1^ h^−1^. This rapid solubilisation of sugars within the first few hours of the process is commonly reported for the hydrolysis of lignocellulose slurries.^26^

The proportion of the soluble sugars that is monomerised likewise increased rapidly in the first 4 to 8 h and then at a much slower rate. This is illustrated by changes in the concentration of glucose which increased rapidly following the addition of enzymes. Monomeric glucose was not detected following pretreatment but concentrations of 7.6 g L^−1^ and 10.3 g L^−1^ were recorded after 4 and 8 h of hydrolysis, respectively. A further 40 h of hydrolysis increased this concentration by only 3.9 g L^−1^. The total concentration of sugars detected at the end of the hydrolysis reaction represents 79% (w/w) of the maximum possible sugar yield.

### Mixing and suspension of DDGS in USD tubes

**Figure 2:**
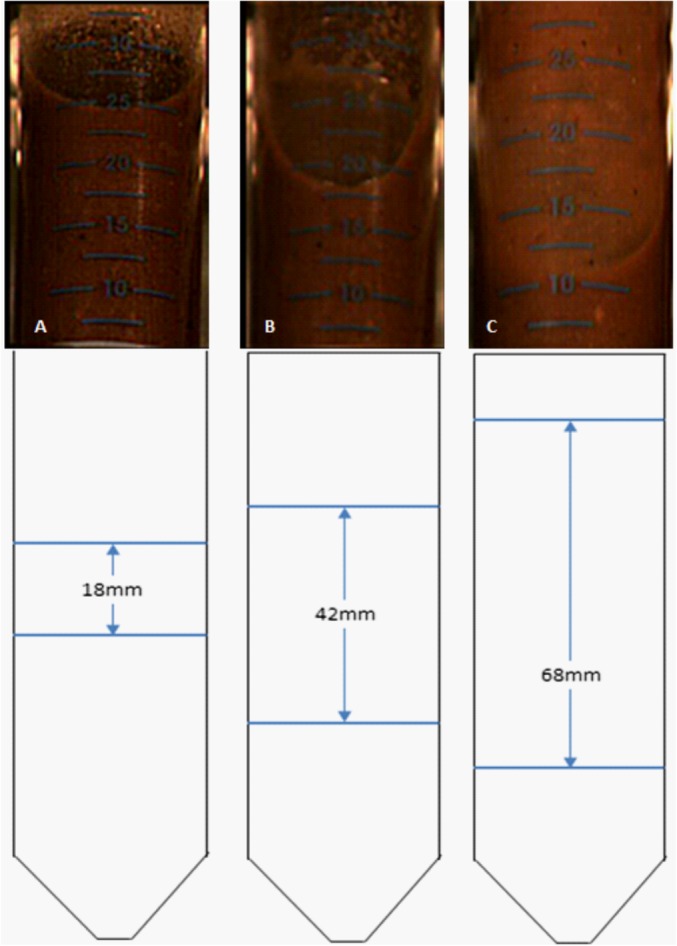
Mixing of 30 mL DDGS hydrolysate, 20% (w/w) total solids loading, in orbitally shaken, vertical USD tubes: (A) 50 rpm; (B) 150 rpm; (C) 250 rpm, along with a schematic diagram showing the position of the interface as well as the height of the meniscus.

It has previously been shown that adequate mixing (solid–liquid suspension) is important for the progression of the enzyme hydrolysis of a lignocellulosic material. Likewise, once there is sufficient mixing to suspend the solids present then additional agitation brings no further benefit.^27^ Figure 2(A)–(C) shows that when the USD tubes are held in a conventional vertical position the depth of the liquid vortex increases as the shaker frequency increases. Visual observations, however, suggested that there may have been a small proportion (<10%) of poorly mixed material remaining in the base of the tubes. In an attempt to alleviate this possibility, mixing was also assessed with the tubes held horizontally at right angles to the shaker bed. Even at a low shaking frequency of 50 rpm there was substantially improved bulk mixing that increased at higher shaking frequencies (data not shown).

Due to this observation, an experiment was set up comparing hydrolysis of DDGS in USD tubes held vertically and horizontally. This indicated that the angle at which the tubes were held did not have a statistically significant effect on the level of sugars solubilised from the DDGS (data not shown). Interpreting this in light of published work suggests that there is adequate mixing when the tubes are held vertically. In line with the earlier observations, increased mixing does not lead to improved performance of the enzyme hydrolysis process. Consequently all further work was carried out with the USD tubes held vertically as this reduces their footprint on the shaker platform.

### Hydrolysis kinetics and yield in USD tubes

**Figure 3:**
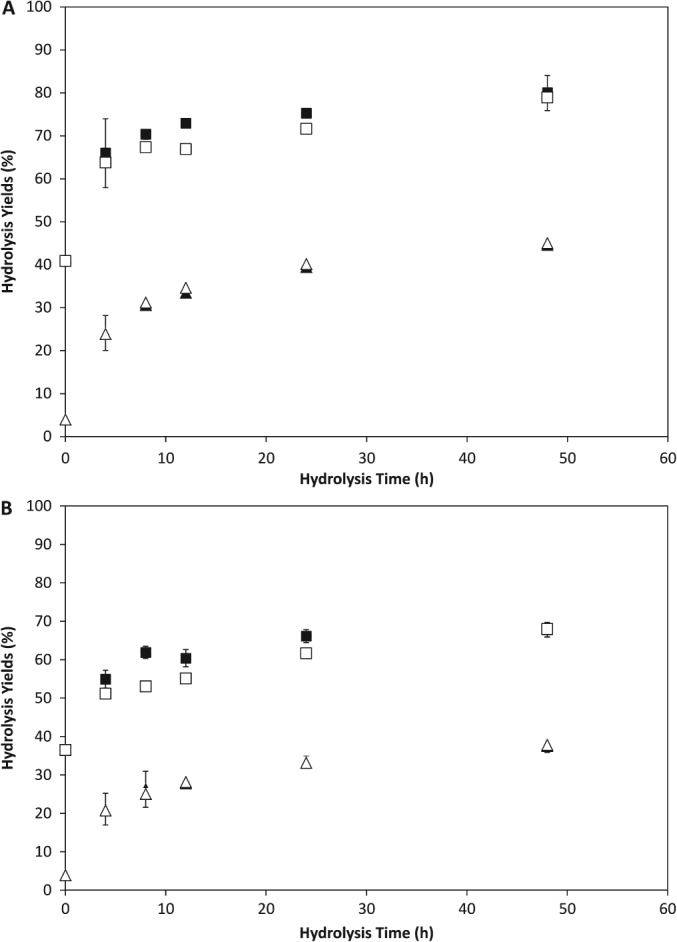
Sugar yields during the hydrolysis of 30 mL DDGS in a USD tube and a 4 L STR at (A) 10% (w/w) total solids loading and (B) 20% (w/w) total solids loading: (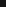) overall yield in USD tubes; (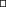) overall yield in STR; (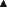) monomer yield in USD tubes; (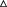) monomer yield in STR. Data from USD tubes is the mean of (n = 3) experiments. Error bars show standard deviation around the mean for USD tubes.

It is highly desirable that any USD model should accurately mimic both the final yields achieved in STRs and the kinetics of the hydrolysis process. An initial indication of such data is crucial in aiding early stage bioprocess design and economic evaluation. In Fig. 3(A) the rates of sugar solubilisation achieved at various time points in the USD tubes are seen to closely mimic the results obtained in the STRs. In the USD tubes the rate of sugar solubilisation in the first 8 h was over 1.5 g L^−1^ h^−1^, dropping to less than 0.1 g L^−1^ h^−1^ in the period between 12 and 48 h. At the end of the hydrolysis there is only a 1% difference in the overall yield between the two scales at 10% DS (w/w), and a 6% difference at 20% DS (w/w) as seen in Fig. 3(B). The comparison between the USD tubes and STRs in terms of the monomerisation of sugars shows essentially no difference between the two scales (ANOVA analysis, P > 0.05).

**Figure 4:**
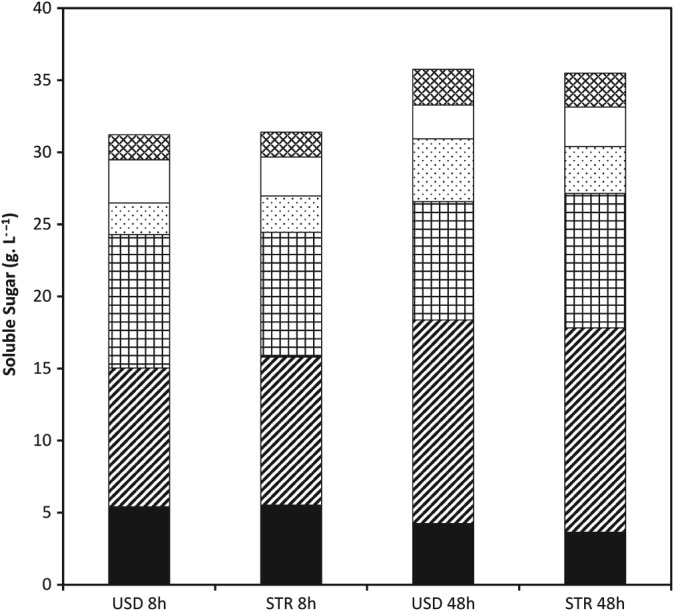
Composition of sugars solubilised at different time points during the hydrolysis of DDGS at 10% (w/w) solids loading in 30 mL USD tubes and 4 L STRs. (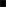 **)** glucose oligomers; (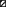) monomeric glucose; (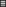) xylose oligomers; (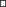) xylose monomer; (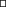) arabinose oligomers; (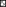) arabinose monomer. Data from USD tubes is the mean of triplicate experiments.

The monomerisation of sugars already solubilised is often thought to resemble more closely simple Michaelis–Menten kinetics.^28^ These results could therefore suggest that there is little difference in mixing between the USD tubes and STRs. It could also be argued, however, that this is simply a reflection of the fact that modern enzyme preparations tend to have an excess of β‐glucosidase present (evidenced by the lack of detectable cellobiose at any point in the time course) that will very rapidly monomerise any cellobiose present. Therefore, as long as the rate of sugar solubilisation is the same in USD tubes and STRs, similar rates of monomerisation will follow as a consequence. In Fig. 4 it can be seen that there is a good degree of similarity in the composition of sugars released during hydrolysis in the USD tubes and STRs. This is true both at the end of the hydrolysis and importantly also at the end of the initial, rapid period of hydrolysis during the first 8 h.

Any USD method must also be able to operate at solids loading representative of those ultimately to be used at large scale. These are likely to be greater than 15% (w/w) in order to generate commercially viable levels of ethanol.^29^ As shown in Fig. 3 the increase in solids loading from 10% DS (w/w) to 20% DS (w/w) led to a small decrease in both the overall and monomeric sugar yield. The general kinetics remained similar however, with between 85% and 88% of the final sugar concentration present after 8 h hours of hydrolysis. The rate of sugar solubilisation in USD tubes was just over 3 g L^−1^ h^−1^ in the first 8 h and 0.2 g L^−1^ h^−1^ between 12 and 48 h. Thus their volumetric rates are almost exactly double those achieved at 10% (w/w) solids content.

The explanation for the fact that volumetric sugar concentrations increase in line with solids content while the hydrolysis yield does not lies in the presence of an increased quantity of insoluble solids. An increasing amount of solids will occupy an increased volume and so while the hydrolysate volume remains constant the volume of water present decreases as solids content is increased. A greater mass of sugar is available to be solubilised into a smaller volume of free water and so the maximum possible concentration of sugar will increase at a greater rate than the hydrolysis dry solids content.^30^

Figure 3(B) shows that a reasonable degree of comparability between USD tubes and STRs is maintained when the solids content during the enzymatic hydrolysis was doubled to 20% (w/w) DS. There is a general trend for the overall yields in the STRs to be somewhat lower than in the USD tubes, although the overall yields from the STRs are less than 10% lower relative to the USD tubes. Such a difference could be explained by a small difference in solids loading. As was the case with the experiments performed at 10% (w/w) solids loading, there was essentially no difference between the two scales in terms of the yield of monomeric sugars achieved (Student's t‐test, *P >* 0.05). Although the mechanism of mechanical agitation in the USD tubes and STRs is different this does not appear to have any significant effect on hydrolysis performance. This is in line with earlier observation that enzyme hydrolysis is a relatively slow process and that additional mixing has no effect on performance once a critical level of mixing sufficient to adequately disperse solids is reached.^27^

### Effect of solids loadings on hydrolysis yields

**Figure 5:**
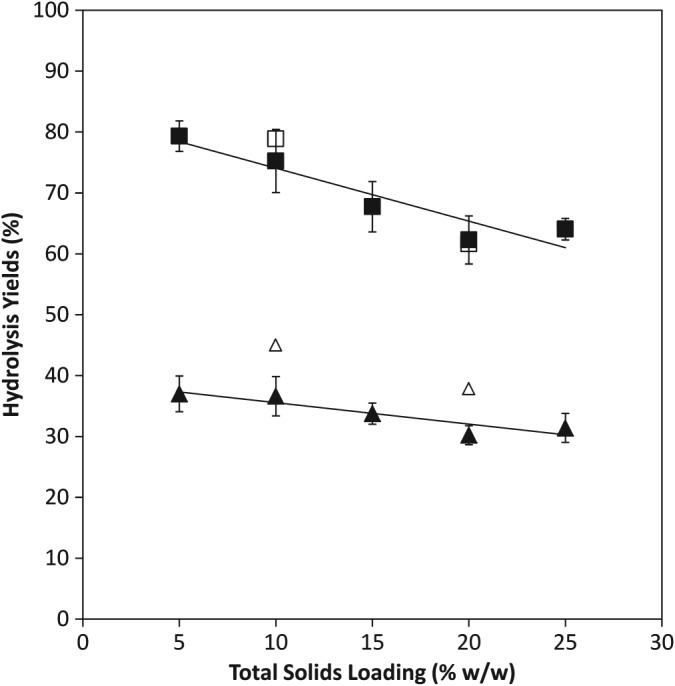
Effect of total solids loading on the release of sugars during the hydrolysis of DDGS at different operating scales: (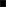) overall yield in USD tubes; (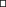) overall yield in STR; (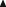) monomer yield in USD tubes; (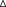) monomer yield in STR. Solid lines fitted by linear regression (R^2^ = 0.88 for overall yield data; 0.84 for monomer yield data). Error bars represent one standard deviation about the mean (n = 3).

Due to the importance of operating at a high solids loading in industrial processes and the differences in performance seen at different solids levels, the influence of solids loading was further investigated. Hydrolyses were carried out in the USD tubes at a range of different initial solids contents with the enzyme loading adjusted in proportion to the level of total dry solids in the hydrolysis. Figure 5 shows a clear, statistically significant, trend for the overall hydrolysis yield achieved at the end of the experiment to decrease as the solids content of the reaction increases (Pearson's Correlation −0.88).^31^ When the solid loading was 5% (w/w), the proportion of the total available sugar that was solubilised was 79%. As the initial solids content of the hydrolysis was raised, there was a linear decrease in the final hydrolysis yield.

When the total dry solids content of the hydrolysis was increased to 20% (w/w) only 62% of the available sugar was solubilised by the end of experiment. This observation is in line with data reported elsewhere on DDGS and other feedstocks.^22^, ^32^ This data obtained from USD tube experiments is also in agreement with data obtained in the 4L STRs especially in terms of the overall yields. As seen in Fig. 5 the decrease in overall yield seen in STRs as the total solids content was increased from 10% (w/w) to 20% (w/w) was of a similar magnitude to the decrease in yield observed in the USD tubes. It therefore seems likely that in STRs the profile for the effect of solids content on hydrolysis yield would be similar to the profile obtained from the USD tubes.

The fact that a linear trend with good correlation was observed for the effect of solids content on hydrolysis yield (Fig. 5) is helpful when trying to obtain as much data as possible from a minimum number of experiments. Using the observed relationship it is possible to extrapolate experimental data obtained at one solids loading to predict the yields that would be achieved at a range of solids loadings. This is likely to be particularly useful in early screening experiments where a very broad range of conditions need to be investigated while using as little resource as possible.

The effect of solids content on the yield of monomeric sugars was less pronounced, although statistically significant, with a Pearson's correlation of −0.82. The yield achieved decreased from 37% at the lowest solids level to 30% at 20% (w/w) DS. Increasing the solids loading from 5% (w/w) to 20% (w/w) led to relative decreases of 18% and 23% for the monomer and overall yields.

The data from Fig. 3 shows that the end of the hydrolysis period there was a difference of 12% between the overall yields of the experiments carried out at 10% (w/w) and 20% (w/w) solids loadings. The difference between the yields at the 8 h time point was 11%, suggesting that this initial period where rapid hydrolysis is occurring may be of particular importance in understanding the role of solids content on the performance of enzymatic hydrolysis of lignocellulose.

### Use of the USD tubes to identify process development priorities

As mentioned above, there is a requirement for bioethanol processes to work at high solids concentrations in order to generate viable levels of ethanol. One commercially relevant application of the USD tubes would therefore be to investigate factors that may be causing the decline in hydrolysis performance at high solids. It has been reported that the performance of enzymatic hydrolysis processes on several different feedstocks is limited by the presence of soluble inhibitors.^33^, ^34^ Other possibilities, such as denaturation of the enzymes, were investigated but found not to affect hydrolysis performance (data not shown). It was therefore decided to use a modification of a washing procedure reported to lead to improved enzymatic hydrolysis through managing the levels of inhibitors present.^35^

**Figure 6:**
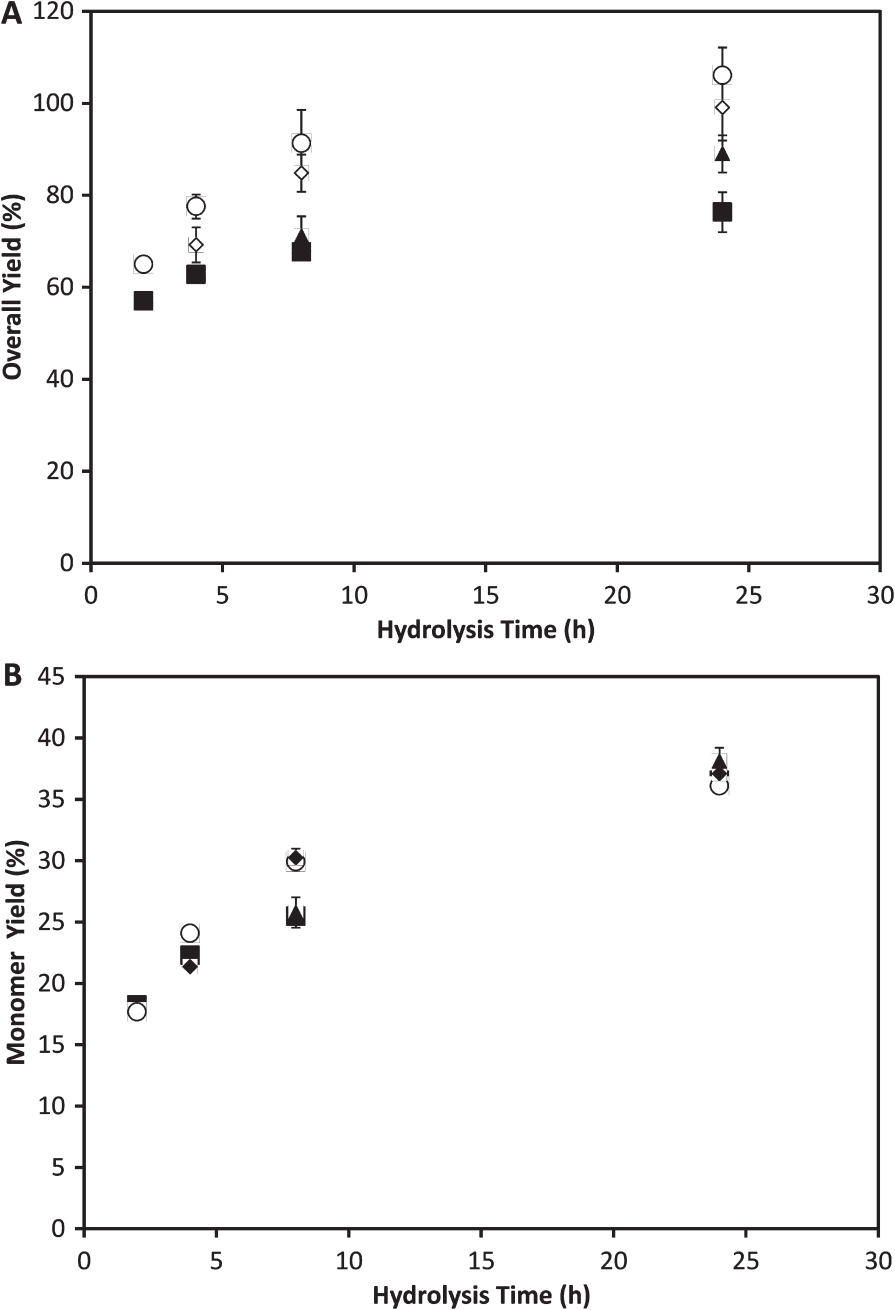
Effect of washing residual solids on the kinetics of (A) total sugar release and (B) monomeric sugar release during DDGS hydrolysis in USD tubes: (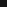) control, solids not washed; (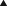) solids washed once during hydrolysis; (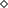) solids washed twice during hydrolysis; (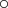) solids washed three times during hydrolysis. Sequence of washing and enzyme addition as described in Table 1. Error bars represent one standard deviation about the mean (n = 3, except for control where n = 2).

Figure 6(A) shows the effect of sequential hydrolysis (washing and re‐suspending the residual solids, before adding fresh enzyme to restart the hydrolysis according to the various protocols described in Table 1). In all cases the total enzyme dosage and overall hydrolysis time was kept constant and previous work had shown that simply splitting the enzyme dosage according to the timings used here gave no benefit to hydrolysis performance (data not shown). It is clear from the USD tube data that there is a significant benefit associated with the sequential hydrolysis procedure used and also that a greater number of washing steps led to a greater increase in overall yield. The final yield was 76% for the control with no washing and this rose to 89% when one washing step was included whilst two or three washing steps led to complete solubilisation of the sugars available from the feedstock.

The hydrolysis process was also quicker, full solubilisation being achieved in less than 24 h compared with the incomplete hydrolysis at 48 h seen in Fig. 3. Rates of sugar solubilisation for the intermediate time points were consistently higher in the washed solids hydrolyses than in the control despite the fact that the concentration of enzyme present at a given time was always higher in the control. Between 2 and 4 h of hydrolysis, for example, almost twice as much sugar was solubilised in the DDGS sample washed three times compared with the control despite the fact that the washed material contained at least a third less enzymes than the control (it was not possible to quantify how much enzyme was lost during the washing procedure and how much remained with the solids). Although a substantial increase in the total levels of sugar solubilised was observed, there was not a corresponding increase in the levels of monomeric sugar produced as demonstrated in Fig. 6(B). This may have been due to the fact that the washing steps meant that the oligomers were not exposed to the enzymes for the full hydrolysis period.

Overall this data suggests that the reason for the improved performance is due to the presence of soluble inhibitors in the hydrolysed DDGS. This is in line with recent publications by other authors which have suggested that a wide variety of chemicals including phenolic compounds and other sugars can affect enzyme hydrolysis performance.^33^, ^34^, ^35^. Further details on the precise nature of these inhibitors can be found in the cited publications. When a washing step is incorporated into the process in order to remove these inhibitors, a given enzyme concentration is able to solubilise significantly more sugars than would otherwise be possible. The USD approach has thus provided insight into where further improvements to the overall process could be made for example focusing on minimising inhibitor creation during the upstream pretreatment and drying steps to minimise the extent of inhibitors while maintaining sugar yields.

## CONCLUSION

This work has established a USD methodology to support bioprocess research and development into lignocellulosic bioethanol production. Using DDGS as a feedstock the USD tubes produced results that were statistically comparable with those achieved in conventional STRs in terms of the final hydrolysis yield, the rate of sugar solubilisation and the effect of the solids content on hydrolysis performance. The USD tubes were then used to examine factors which may have been limiting process performance. It was found that soluble inhibitors were preventing the enzymes used from fully solubilising the available sugars thus providing a focus for further process development.
